# Choroidal changes after vitrectomy performed for macular hole retinal detachment

**DOI:** 10.1007/s10384-025-01218-y

**Published:** 2025-06-18

**Authors:** Hirotaka Sumida, Ikuko Umeda, Takayuki Baba

**Affiliations:** Department of Ophthalmology and Visual Science, Chiba University Graduate School of Medicine, 1-8-1 Inohana, Chuo-ku, Chiba, 260-0856 Japan

**Keywords:** Choroidal thickness, High myopia, Macular hole retinal detachment, Vitrectomy, Optical coherence tomography

## Abstract

**Purpose:**

To investigate changes in the choroidal area (CA), luminal area (LA), stromal area (SA), choroidal vascularity index (CVI), and choroidal thickness (CT) before and after vitrectomy in eyes with macular hole retinal detachment (MHRD).

**Study design:**

Retrospective observational study.

**Methods:**

We measured the best-corrected visual acuity (BCVA), intraocular pressure (IOP), CA, LA, SA, CVI, and CT preoperatively and 1, 3, and 6 months postoperatively in 10 eyes with MHRD. CA was measured within a 3-mm-wide area around the fovea in the horizontal and vertical images. LA and SA were quantified using the Niblack method, and CVI was calculated as the ratio of LA to CA. CT was measured at the subfovea and at 1 and 3 mm vertically and horizontally away from the fovea.

**Results:**

BCVA improved significantly at 1 and 3 months postoperatively (*P* = 0.036 and 0.016). IOP remained stable. CA and LA decreased significantly 6 months postoperatively in both the horizontal (*P* = 0.002 and 0.014) and vertical sections (*P* = 0.006 and 0.002). SA remained stable. CVI reduced significantly at 1 month horizontally and at 3 months vertically (both *P* = 0.027). CT decreased significantly in the subfovea at 3 and 6 months postoperatively (*P* = 0.027 and 0.020, respectively). Significant reductions were also observed at 1 mm nasal, temporal, and superior regions (*P* = 0.014, 0.014, and 0.004) and at 2 mm temporal and superior regions 1 month postoperatively (*P* = 0.020 and 0.014).

**Conclusion:**

Choroidal thinning was observed after vitrectomy in eyes with MHRD, driven by a reduction in the luminal area.

## Introduction

The number of myopia patients is rapidly increasing worldwide, particularly in East Asia. It is estimated that by 2050, the number of myopic patients will reach 5 billion, with 1 billion having high myopia greater than −5.0 diopters [1]. High myopia occurs due to elongation of the axial length, and instigates complications, such as myopic maculopathy and glaucoma [2, 3]. These complications potentially lead to irreversible severe vision loss, and its prevalence increases with the degree of myopia. Therefore, prevention of high myopia and treatment of its complications are important.

Macular hole-retinal detachment (MHRD) can cause severe vision loss in patients with high myopia. The development process involves traction by the posterior vitreous cortex, epiretinal membrane (ERM), internal limiting membrane (ILM), and retinal vasculature, leading to macular schisis, progression to foveal detachment, and further traction leading to the formation of macular hole, finally resulting in the development of MHRD. Posterior staphyloma, frequently observed in eyes with severe axial elongation and high myopia, is associated with the development of MHRD [3].

Furthermore, the choroid nourishes and supplies oxygen to the outer layers of the retina and retinal pigment epithelium (RPE) [4]. Therefore, the choroid is associated with the pathophysiology of several ocular diseases, including myopia. Recent studies suggest that choroidal thinning is associated with the progression of myopic maculopathy [5, 6]. Additionally, macular atrophy occurs in 30% of the cases after vitrectomy for MHRD [7]. Based on these reports, choroidal thinning is expected after MHRD surgery; however, choroidal thickness in post-MHRD patients is not clearly understood. In this study, we measured and evaluated the differences between preoperative and postoperative choroidal area (CA), luminal area (LA), stromal area (SA), choroidal vascularity index (CVI), and choroidal thickness (CT) in eyes with MHRD as observed on enhanced-depth imaging optical coherence tomography (EDI-OCT) images. CVI is an innovative parameter introduced to evaluate the status of choroidal blood flow [8]. CVI is calculated from EDI-OCT images through a process involving digital binarization and quantification.

## Methods

### Subjects and study design

We conducted a retrospective study including 10 patients (10 eyes) with macular hole retinal detachment (MHRD) who underwent vitrectomy at Chiba University Hospital between January 2015 and November 2019. Exclusion criteria included cases in which preoperative observation of the choroid by OCT was difficult and cases with proliferative vitreoretinopathy, media opacities, and choroidal detachment or hypotony.

### Surgical procedures

All the surgery was performed by a single experienced retinal surgeon (TB). Cataract surgery was performed if a visually significant cataract was present and could interfere with posterior segment manipulation. A conventional 3-port vitrectomy was performed using a 25-gauge system. Triamcinolone acetonide was injected into the vitreous cavity to aid in the visualization of the vitreous cortex, which was then gently removed from the posterior retina using forceps. Following subretinal fluid drainage through the macular hole, ILM was stained with 0.025% brilliant blue G (BBG). ILM peeling was performed using end-gripping forceps, initiated approximately 3-disc diameters from the fovea and performed gently in a circular fashion. One eye underwent ILM peeling, while 9 cases underwent inverted ILM insertion. After the ILM manipulation, a fluid–air exchange was performed. Three cases were followed by injection of expansion gas (SF₆) with a volume of 1.0–1.5 cc. Postoperatively, patients were instructed to maintain a face-down position for one week until the macula could be viewed by OCT.

### Examinations

**Fig. 1:**
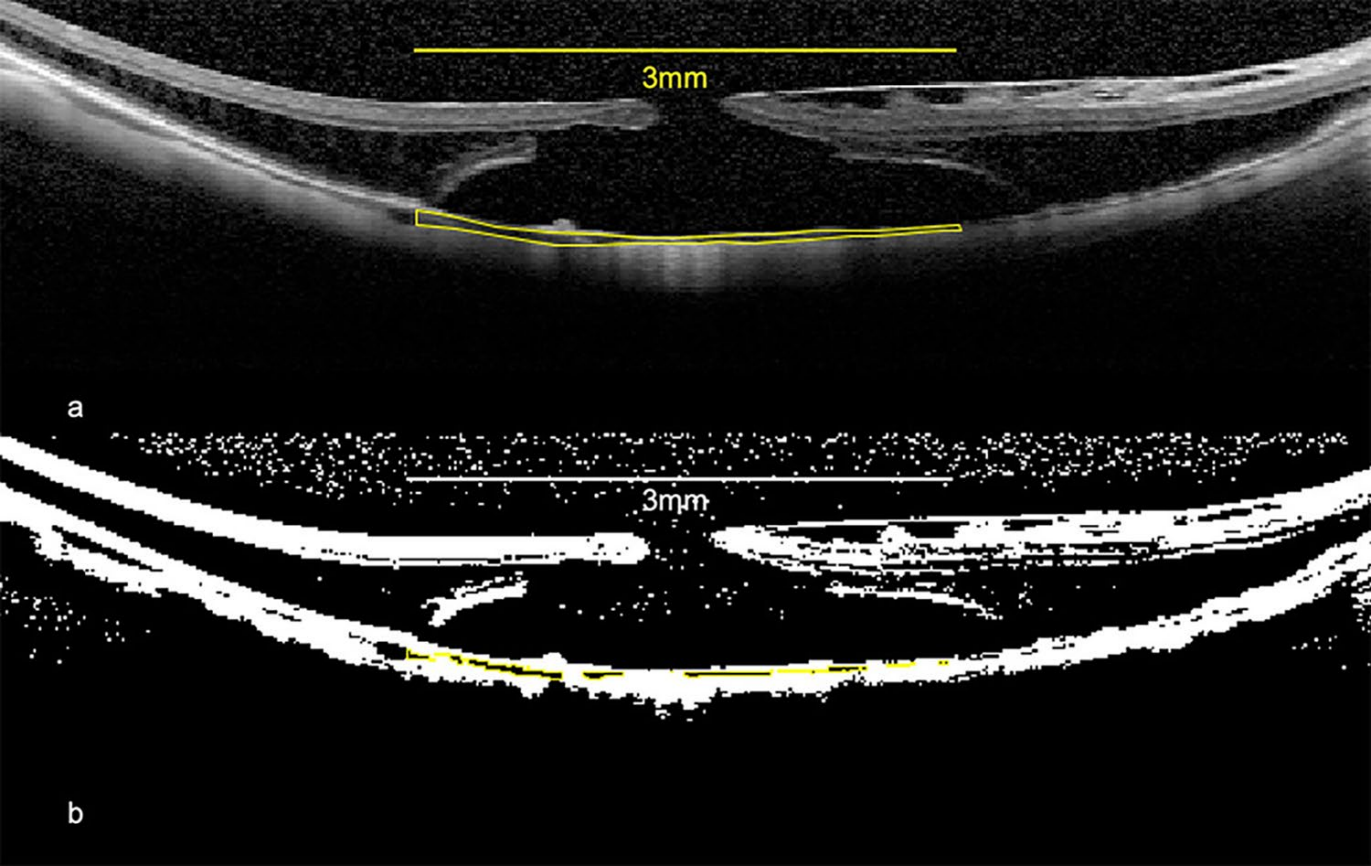
An EDI OCT image and binary image of a MHRD eye. CA was measured within an area 3 mm wide around the fovea (**a**). An EDI OCT Image was converted to a binary image using software Image J. The area surrounded by a yellow line was excised. The white and dark areas correspond to SA and LA (**b**). *EDI-OCT* enhanced-depth imaging optical coherence tomography; *MHRD* macular hole retinal detachment; *CA* choroidal area; *LA* luminal area; *SA* stromal area

We measured the best-corrected visual acuity (BCVA), intraocular pressure (IOP), CA, LA, SA, CVI, and CT preoperatively and at 1, 3, and 6 months postoperatively using EDI-OCT images. The EDI-OCT images were obtained using spectral-domain OCT (Heidelberg Spectralis OCT) and analyzed using open-access imaging software Image J (Version 1.5, National Institute of Health). We conducted all OCT examinations between 11:00 AM and 2:00 PM to reduce the influences of diurnal variations on the choroid. The choroid was defined as the area between the outer border of the retinal pigment epithelium and the choroidal–scleral interface. CA was measured within a 3-mm-wide area around the fovea in horizontal and vertical images. LA and SA were quantified using the Niblack method (Fig. 1) [9]. CVI was defined as the proportion of LA to CA. CT was measured at the subfovea and 1 and 3 mm vertically and horizontally away from the fovea. All choroidal measurements were performed by two experienced examiners (HS and IU), and the average values were adopted for further analysis.

### Statistical analysis

Using the Wilcoxon signed-rank test, we determined the significance of differences in BCVA, IOP, CA, LA, SA, CVI and CT before surgery and at 1, 3, and 6 months after surgery. All statistical analyses were performed using EZR [10].

### Ethical considerations

The study was conducted in accordance with the ethical guidelines of the Declaration of Helsinki, with the approval of the Chiba University Observational Research Institutional Review Board (Approval Number HK202403-15). 

## Results

### Demographic and clinical characteristics

Ten eyes from 10 patients with MHRD were evaluated in this study (Table 1). All the patients were women. The mean age was 69.6 ± 8.4 years (range: 57–82). The mean axial length was 28.8 ± 1.1 mm. Based on the patients’ symptoms, the duration of MHRD was estimated in 8 cases, with an average of 2.5 ± 1.4 months. The size of macular hole was 374 ± 243 µm. ERM was observed in 9 cases, macular schisis and posterior staphyloma in all 10 cases. Dome-shaped macula was not observed in any of the cases. Seven eyes underwent combined 25-gauge vitrectomy and cataract surgery, whereas three eyes underwent vitrectomy alone. Surgery time was 48.8 ± 14.4 min, with 30.9 ± 29.2 min of macular tissue manipulation.

**Table 1:** Demographic and clinical characteristics of the patients with MHRD (*n*=10)

Age (years, mean±SD)	69.6±8.4
Operated eye (right/left)	5/5
Sex (male/female)	0/10
Lens status (phakia/pseudophakia)	7/3
BCVA (logMAR, mean±SD)	0.68±0.36
Axial length (mm, mean±SD)	28.8±1.1
Duration of symptoms	
Duration known	8
The duration of MHRD (months, mean±SD)	2.5±1.4
Size of macular hole (μm, mean±SD)	374±243
Presence of ERM	9
Presence of schisis	10
Presence of posterior staphyloma	10
Presence of dome-shaped macula	0
Combined cataract extraction	70%
Tamponade (Air/SF6)	7/3
Vital dye (BBG)	10
Surgery time (min, mean±SD)	48.8±14.4
Time for manipulation of macular tissue (mean±SD)	30.9±29.2
IOP (mmHg)	
Baseline	13.1±1.8
1 m	13.5±2.2
3 m	12.7±2.4
6 m	13.0±2.6
BCVA (logMAR)	
Baseline	0.68±0.36
1 m	0.52±0.35
	**(*p*=0.036)**
3 m	0.46±0.41
	**(*p*=0.016)**
6 m	0.48±0.50
Newly detected MA during 6 months	2
Period until detection of MA	2 m, 4 m

Values are presented as n or mean ± SD. The numbers within parentheses denote* P* values derived using the Wilcoxon signed-rank test to compare the baseline data with postoperative data.

The numbers within parentheses denote* P* values derived using the Wilcoxon signed-rank test to compare baseline data with postoperative data.

*BCVA* best-corrected visual acuity; *logMAR* logarithmic angle of resolution; *ERM* epiretinal membrane; *BBG* Brilliant Blue G; *MA* macular atrophy; *m* months (s). Boldface indicates statistical significance.

**Fig. 2:**
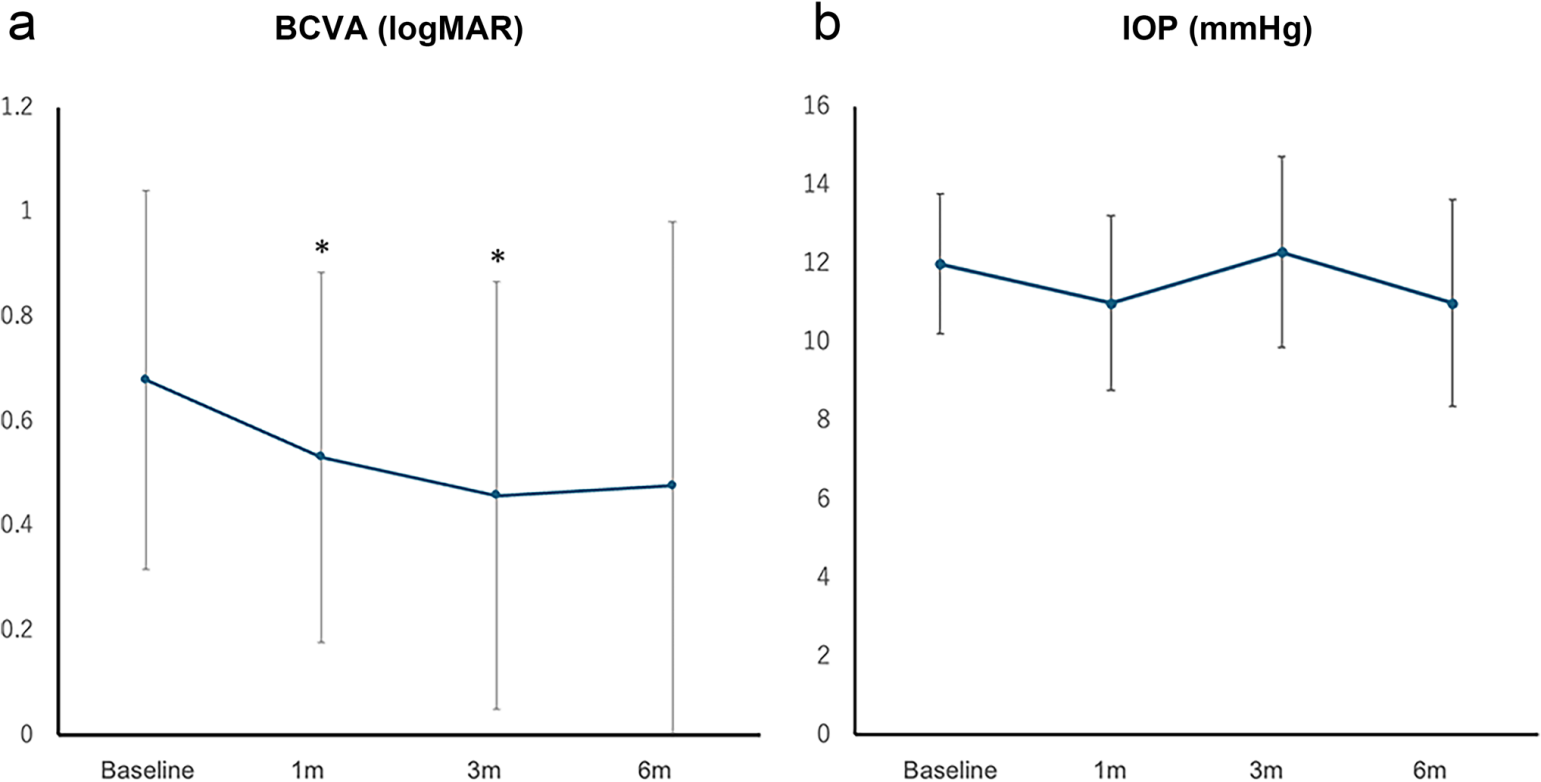
The change in BCVA and IOP after the surgery. **a** The BCVA significantly improved at 1 and 3 months postoperatively (*P* = 0.036, 0.016, respectively). **b** The IOP showed no significant change. Asterisks indicate a significant difference when compared with baseline (*P < 0.05). *P*-values were derived using the Wilcoxon signed-rank test to compare the baseline data and postoperative data. *BCVA* best-corrected visual acuity; *IOP* intraocular pressure; *m* months (s)

The changes in BCVA and IOP are shown in Fig. 2. BCVA improved from 0.68 ± 0.36 logMAR at baseline to 0.48 ± 0.50 logMAR at 6 months after surgery (*P* = 0.066). A significant improvement was observed in BCVA at 1 and 3 months (*P* = 0.036 and 0.016, respectively). No significant changes were observed in IOP between the baseline and postoperative periods. Two eyes (20%) developed new macular atrophy within 6 months postoperatively, detected at 2 and 4 months, respectively.

### Accuracy of choroidal measurements

The choroidal measurements demonstrated excellent reproducibility, with consistently high intraclass correlation coefficient (ICC). For example, the ICC for CA in the horizontal sections was consistently high, with CA showing 0.989 (95% CI, 0.955–0.997) at baseline and 0.986 (95% CI, 0.949–0.997) at 1 week postoperatively.

### Changes in choroidal area, luminal area, stromal area, and choroidal vascularity index

**Fig. 3:**
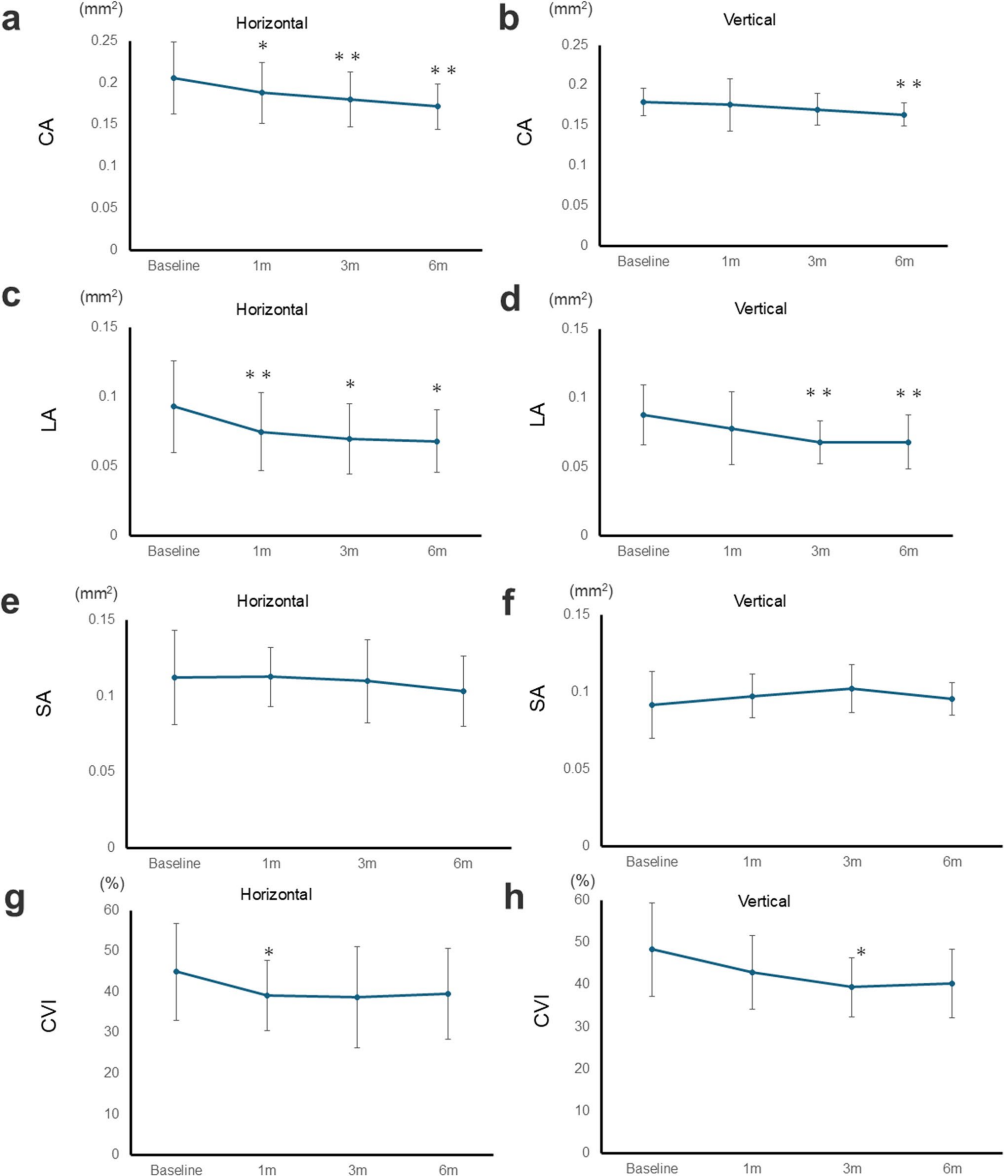
Baseline and postoperative choroidal area and luminal area over 6 months following surgery for MHRD. **a** In the horizontal section, CA significantly decreased at 1, 3, 6 months (*P* = 0.049, 0.010, 0.002, respectively). **b** In the vertical section, CA significantly decreased at 6 months (*P* = 0.006). **c** In the horizontal section, LA significantly decreased at 1, 3, 6 months (*P* = 0.004, 0.020, 0.014, respectively). **d** In the vertical section, LA significantly decreased at 3 and 6 months (*P* = 0.020, 0.014, respectively) **e**, **f** In the stromal area, slight fluctuations were shown over time with no significant changes. **g** In the horizontal section, CVI significantly decreased at 1 month (*P* = 0.027). **h** In the vertical section, CVI significantly decreased at 3 months (*P* = 0.027). Asterisks indicate a significant difference compared to the baseline values (* *P* < 0.05, ** *P* < 0.01). *P*-values were derived using the Wilcoxon signed-rank test to compare baseline data with postoperative data. *CA* choroidal area; *LA* luminal area; *SA* Stromal area; *CVI* choroidal vascularity index; *m* months (s)

The changes in CA, LA, SA and CVI are shown in Fig. 3, Table 2.

**Table 2:** Temporal change of CA, LA, SA, and CVI (mean ± standard deviation) following surgery for MHRD

	Baseline	1 m	3 m	6 m
CA (mm^2^)				
Horizontal	0.206 ± 0.0435	0.189 ± 0.0361	0.180 ± 0.0323	0.171 ± 0.0268
		**(0.049)**	**(0.0098)**	**(0.0020)**
Vertical	0.179 ± 0.0174	0.175 ± 0.0329	0.170 ± 0.0199	0.164 ± 0.0139
		(0.70)	(0.23)	**(0.0059)**
LA (mm^2^)				
Horizontal	0.093 ± 0.0330	0.075 ± 0.0281	0.070 ± 0.0253	0.0682 ± 0.0225
		**(0.0039)**	**(0.020)**	**(0.014)**
Vertical	0.0877 ± 0.0215	0.0781 ± 0.0264	0.0677 ± 0.0155	0.0681 ± 0.0193
		(0.084)	**(0.0020)**	**(0.0020)**
SA (mm^2^)				
Horizontal	0.113 ± 0.0311	0.113 ± 0.0194	0.110 ± 0.0274	0.103 ± 0.0230
		(0.96)	(0.92)	(0.084)
Vertical	0.0915 ± 0.0217	0.0974 ± 0.0141	0.102 ± 0.0156	0.0955 ± 0.0107
		(0.63)	(0.32)	(0.56)
CVI (%)				
Horizontal	44.9 ± 11.8	39.2 ± 8.66	38.7 ± 12.4	39.5 ± 11.1
		**(0.027)**	(0.064)	(0.11)
Vertical	48.4 ± 11.1	43.0 ± 8.73	39.4 ± 7.08	40.4 ± 8.19
		(0.13)	**(0.027)**	(0.11)

Numbers within parentheses denote* P* values derived using the Wilcoxon signed-rank test to compare the baseline data with postoperative data.

*CA* choroidal area; *LA* luminal area; *SA* stromal area; *CVI* choroidal vascularity index; *m* month(s)

Boldface indicates statistical significance

CA significantly decreased in both the horizontal and vertical sections. In the horizontal section, CA was 0.206 ± 0.044 mm^2^ at baseline. At 1 month, it decreased to 0.189 ± 0.036 mm^2^ (*P* = 0.049), it further decreased to 0.180 ± 0.032 mm^2^ at 3 months (*P* = 0.010), and it was 0.171 ± 0.027 mm^2^ at 6 months (*P* = 0.002). In the vertical section, CA was 0.179 ± 0.017 mm^2^ at the baseline. At 6 months visit, it decreased to 0.164 ± 0.014 mm^2^ (*P* = 0.006).

Similarly, LA significantly decreased in both sections. In the horizontal section, LA was 0.093 ± 0.033 mm^2^ at the baseline. At 1 month, it decreased to 0.075 ± 0.028 mm^2^ (*P* = 0.004), it further decreased to 0.070 ± 0.025 mm^2^ at 3 months (*P* = 0.020), and it was 0.068 ± 0.023 mm^2^ at 6 months (*P* = 0.014). In the vertical section, the LA was 0.088 ± 0.022 mm^2^ at baseline. At the 3 months visit, it decreased to 0.068 ± 0.016 mm^2^ (*P* = 0.002), and it was 0.068 ± 0.019 mm^2^ at 6 months (*P* = 0.002).

SA remained stable in both the horizontal and vertical sections. In the horizontal section, SA was 0.113 ± 0.031 mm^2^ at baseline, and 0.103 ± 0.023 mm^2^ at 6 months. Similarly, in the vertical section, SA was 0.091 ± 0.022 mm^2^ at baseline, and 0.095 ± 0.011 mm^2^ at 6 months.

CVI showed significant reductions in both sections. In the horizontal section, CVI was 45.0 ± 11.8 at baseline, decreased significantly to 39.2 ± 8.7 at 1 month (*P* = 0.027), and remained stable at 39.5 ± 11.1 at 6 months. In the vertical section, CVI was 48.4 ± 11.1 at baseline, significantly decreased to 39.4 ± 7.1 at 3 months (*P* = 0.027), and remained stable at 40.4 ± 8.2 at 6 months.

### Postoperative changes in choroidal thicknesses

**Fig. 4:**
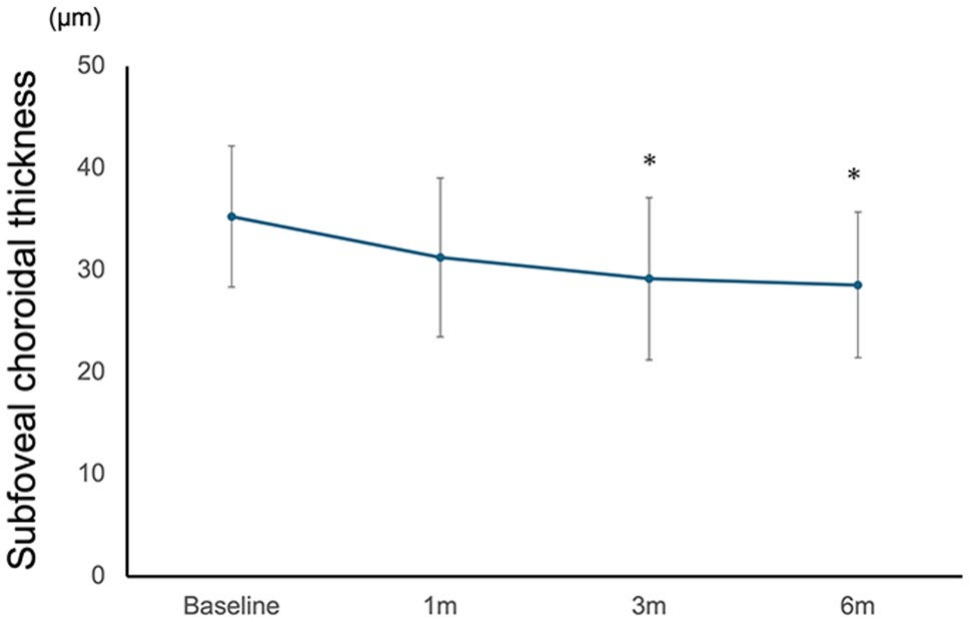
Baseline and postoperative subfoveal choroidal thickness over 6 months following surgery for MHRD. The subfoveal choroidal thickness significantly decreased at 3, 6 months postoperatively (*P* = 0.027, 0.020, respectively). Asterisks indicate a significant difference when compared with baseline data (* *P* < 0.05). *P* values were derived using the Wilcoxon signed-rank test for baseline and postoperative visits. *m* month(s)

**Fig. 5:**
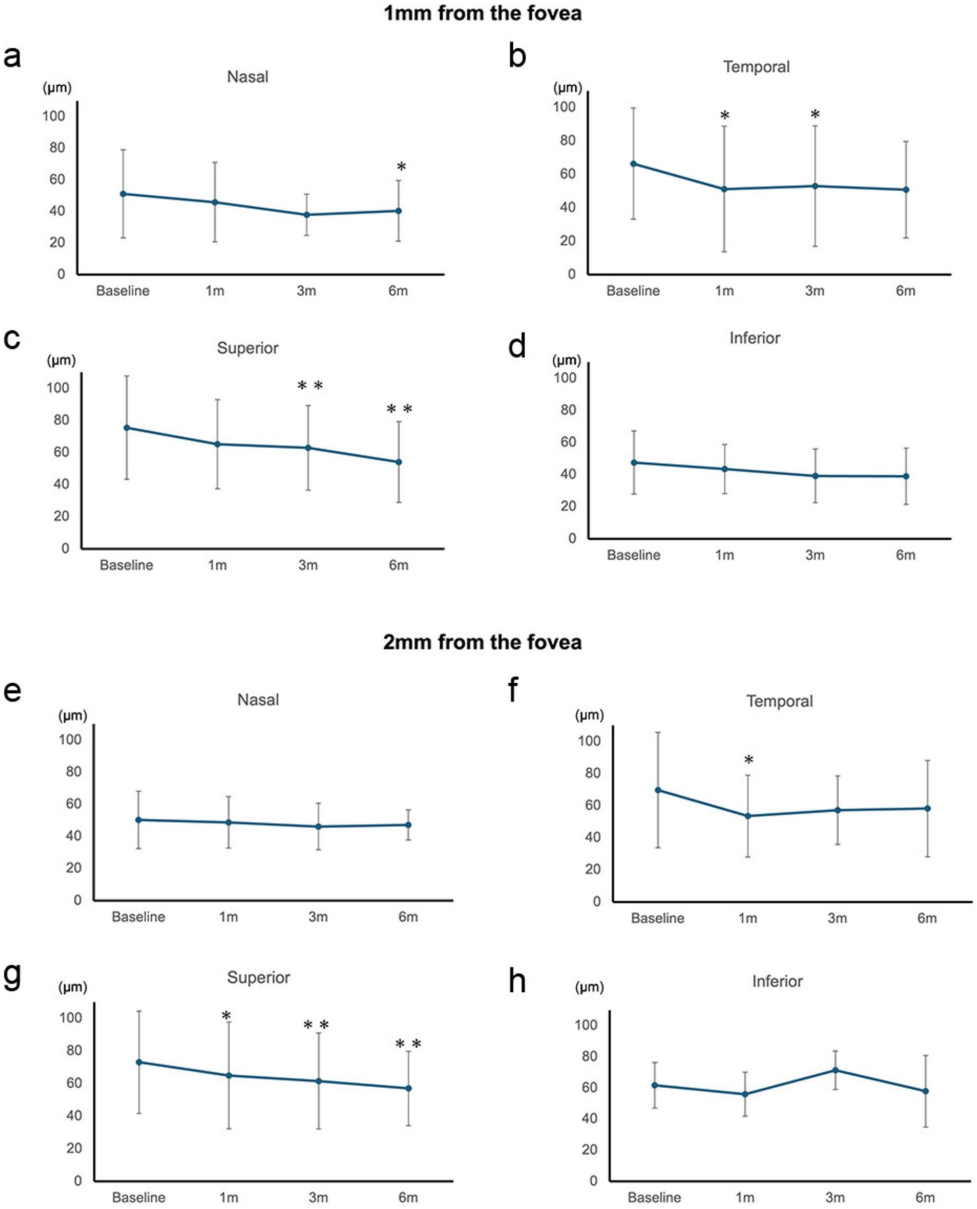
The change of CT after the surgery at multiple locations, including areas at 1 mm and 2 mm from the fovea. **a**–**d** At 1 mm away from the fovea. **a** Nasal area: A slight decrease was observed over time, with a significant difference at 6 months (*P* = 0.014). **b** Temporal area: Significant reductions were observed at 1 and 3 months (*P* = 0.014, 0.014, respectively). **c** Superior area: A steady decrease was observed over time, with significant differences at 3 and 6 months (*P* = 0.010, 0.004, respectively). **d** Inferior area: Minimal changes were observed over time, with no significant differences. **e**–**h** At 2 mm away from the fovea. **e** Nasal area: The CT remained consistent over time. **f** Temporal area: A significant reduction was observed at 1 month (*P* = 0.020), with no further significant changes. **g** Superior area: Steady reductions were observed at 1, 3, 6 months (*P* = 0.014, 0.006, 0.010, respectively). **h** Inferior area: Slight fluctuations were observed over time with no significant changes. Asterisks indicate a significant difference compared to baseline data (* *P* < 0.05, ** *P* < 0.01). *P*-values were derived using the Wilcoxon signed-rank test to compare baseline data with postoperative data. *m* month(s)

The postoperative changes in choroidal thickness at the subfovea and at multiple locations, including regions at 1 and 2 mm from the fovea, are shown in Figs. 4 and 5.

Foveal thickness and CT decreased at 3 and 6 months (*P* = 0.027 and 0.020, respectively, Table 3).

**Table 3:** Temporal change of choroidal thickness (mean ± standard deviation) following surgery for MHRD.

Location	Choroidal thickness (µm)			
	Baseline	1 m	3 m	6 m
Subfovea	35.3 ± 6.9	31.2 ± 7.8	29.2 ± 7.9	28.6 ± 7.1
		(0.280)	**(0.027)**	**(0.020)**
Temporal, 1 mm	66.4 ± 33.3	51.2 ± 37.6	52.9 ± 36.0	50.9 ± 28.9
		**(0.014)**	**(0.014)**	(0.064)
Temporal, 2 mm	69.7 ± 35.9	53.5 ± 25.4	57.2 ± 21.4	58.2 ± 30.0
		**(0.020)**	(0.110)	(0.230)
Nasal, 1 mm	51.0 ± 27.9	45.9 ± 25.2	37.9 ± 13.1	40.4 ± 19.2
		(0.920)	(0.110)	**(0.014)**
Nasal, 2 mm	50.3 ± 17.9	48.8 ± 16.0	46.1 ± 14.6	47.1 ± 9.3
		(0.700)	(0.380)	(0.850)
Superior, 1 mm	75.5 ± 32.2	65.1 ± 27.8	62.3 ± 26.4	54.1 ± 25.1
		(0.190)	**(0.010)**	**(0.004)**
Superior, 2 mm	73.1 ± 31.4	65.0 ± 32.8	61.6 ± 29.6	57.0 ± 22.8
		**(0.014)**	**(0.006)**	**(0.010)**
Inferior, 1 mm	47.5 ± 19.7	43.5 ± 15.3	39.2 ± 16.8	38.9 ± 17.5
		(0.320)	(0.130)	(0.084)
Inferior, 2 mm	61.7 ± 14.7	56.0 ± 14.2	71.3 ± 12.4	57.9 ± 12.3
		(0.560)	(0.280)	(0.380)

Numbers within parentheses denote *P* values derived using the Wilcoxon signed-rank test to compare the baseline data with postoperative data.

*m* month(s)

Boldface indicates statistical significance

CT of the temporal area 1 mm away from the fovea showed a significant decrease 1 month after surgery (*P* = 0.014).

CT of areas 2 mm away from the fovea showed a significant decrease 1 month after surgery at the temporal and superior locations (*P* = 0.020 and 0.014, respectively).

The CT at superior-1 and 2 mm locations further reduced from 75.5 ± 32.2 µm and 73.1 ± 31.4 µm to 62.3 ± 26.4 µm and 61.6 ± 29.6 µm (*P* = 0.010 and 0.006) and decreased even more to 54.1 ± 25.1 µm and 57.0 ± 22.8 µm (*P* = 0.004 and 0.010).

## Discussion

In this study, we evaluated choroidal measurements up to 6 months after vitrectomy for MHRD. CA, LA and CVI decreased after surgery, and CT also decreased, with the most significant reduction observed in the superior choroid. In contrast, SA remained stable. These changes suggest that choroidal thinning is primarily driven by a reduction in choroidal vascular portion.

The cause of choroidal thinning after surgery remains unclear; however, several factors may be involved.

First, intravitreal gas may compress the choroid. Considering that the gas bubble persists in the eye for approximately 2 weeks, the remarkable decrease in superior CT compared to inferior CT in the early post-operative period may be attributed to gas tamponade. The possibility of choroidal compression due to gas tamponade is also suggested in a previous report evaluating CT after vitrectomy for ERM and macular holes in eyes without high myopia [11].

Second, the combined toxic effects of brilliant blue G (BBG) and endoillumination may cause progressive damage to the RPE [12]. In normal eyes, the RPE secretes vascular endothelial growth factor (VEGF) to maintain choroidal vasculature [13]. In contrast, loss of VEGF secretion by the RPE leads to choroidal thinning [14]. Prolonged retinal detachment can lead to changes such as migration and stratification of the RPE. In the case of MHRD, which often persists for a long duration, surgical intervention may cause further damage to the RPE cells.

Considering that the postoperative IOP remained unchanged from the baseline in our cases, it seems unlikely that a high IOP caused choroidal perfusion disturbance and choroidal thinning. Although increased oxygen partial pressure in the vitreous cavity after surgery may cause vasoconstriction and result in choroidal perfusion disturbances or choroidal thinning, the high autoregulatory ability of choroidal blood vessels makes it unlikely for these factors alone to account for choroidal thinning.

**Fig. 6:**
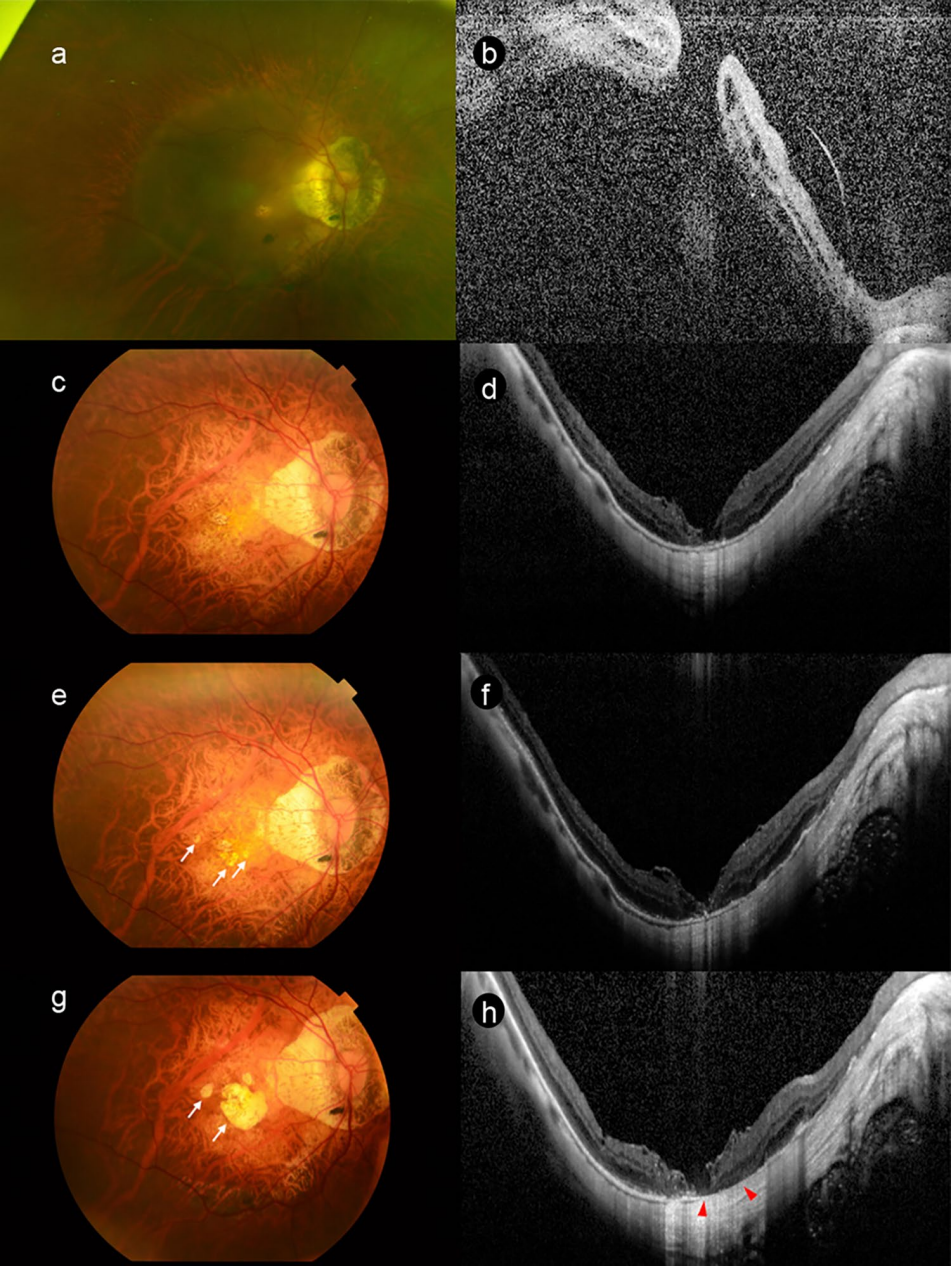
Development of macular atrophy and choroidal thinning after surgery for MHRD. **a** Preoperative fundus of a 77-year-old-man with axial length of 29.72 mm showed MHRD. The BCVA was 0.06. **b** Horizontal section of a preoperative OCT image showed MHRD. Significantly high height of retinal detachment hindered the observation of choroid. **c** One month after the phaco and vitrectomy with BBG-assisted ILM peeling and using an inverted ILM flap, MHRD was resolved. The BCVA is 0.06. **d** Horizontal OCT image at 1 month after the surgery. Subfoveal choroidal thickness (SFCT) was 19.5 µm. **e** Six months after the surgery. Small whitish lesions can be seen (arrows). The BCVA is 0.10. **f** Horizontal OCT image at 6 months after the surgery showed the progression of choroidal thinning. SFCT was 11.8 µm. **g** At 42 months after the surgery, the MA appeared enlarged (arrows). The BCVA was 0.03. **h** Horizontal OCT image taken at 42 months after the surgery showed the defect of RPE and choroid (between arrowheads). SFCT decreased further to 6.5 µm. *SFCT* subfoveal choroidal thickness; *m* month(s)

In this study, choroidal thinning was observed during the early postoperative period and this change was progressive. In the case shown in Fig. 6, which was followed up for 42 months after MHRD surgery, choroidal thinning preceded the development of macular atrophy. During the 6 months follow-up, we observed macular atrophy that began as a small whitish lesion in 2 out of 10 eyes. Fang et al. [7] report that 30% of MHRD eyes developed fovea-centered macular atrophy, initially observed as a small, isolated whitish lesion at the fovea in the early postoperative period. Taken together, these findings suggest that early postoperative choroidal thinning may lead to the formation of small whitish lesions that eventually progress into macular atrophy.

Our study has several limitations. First, the sample size is small (10 patients). MHRD is a relatively rare disease, and OCT observation is not always possible because of media opacity and extensive retinal detachment. Second, choroidal image acquisition using SD-OCT was difficult in cases with extensive retinal detachment, which exceeded the measurement range of OCT, and these cases were not included in our study. Third, accurate measurements were challenging because some of the obtained OCT images were of poor-quality owing to extremely long axial lengths, and we had to evaluate an extremely thin choroid. In our study, two experienced examiners measured the choroid to address these issues. Moreover, the observation period was relatively short at six months, and no relationship between atrophy progression and choroidal thickness was observed over a longer period. Wu et al. observed the changes in the progression of myopic maculopathy associated with visual deterioration from 12 to 36 months post MHRD-surgery [15]. Further studies with longer observation periods are required to confirm these findings.

In conclusion, our study demonstrates choroidal thinning during the early postoperative period in eyes with MHRD, primarily driven by a reduction in choroidal vascular portion. Thus, choroidal thinning may cause chorioretinal atrophy. Therefore, long-term follow-up that includes choroidal evaluation is essential.
